# lncRNA LEF1-AS1 Acts as a Novel Biomarker and Promotes Hypopharyngeal Squamous Cell Carcinoma Progression and Metastasis by Targeting the miR-221-5p/GJA1 Axis

**DOI:** 10.1155/2022/3881310

**Published:** 2022-03-25

**Authors:** Junda Fan, Cheng Wang, Xingyou Zhai, Jianhui Li, Jun Ju, Yuying Zhu, Shikang Zheng, Nan Ren, Bangqing Huang, Xinying Jiang, Yingli Xie, Kai Zhao, Mingbo Liu

**Affiliations:** ^1^Medical School of Chinese PLA, Beijing, China; ^2^Department of Otolaryngology Head and Neck Surgery, Hainan Hospital of Chinese PLA General Hospital, Sanya, China; ^3^School of Basic Medical Sciences, Weifang Medical University, Weifang, China; ^4^School of Clinical Medicine, Weifang Medical University, Weifang, China; ^5^The Second School of Clinical Medicine, Southern Medical University, Guangzhou, China

## Abstract

Hypopharyngeal squamous cell carcinoma (HSCC) is highly malignant and extremely aggressive, making it one of the worst prognoses among all kinds of head and neck squamous cell carcinoma (HNSCC); therefore, gaining insight into molecular mechanisms of HSCC is of profound significance. In the current manuscript, we revealed the elevated expression of long noncoding RNA (lncRNA) LEF1-AS1 in HNSCC which was associated with the poor prognosis by bioinformatic analysis. Moreover, we noticed that LEF1-AS1 dramatically accelerated the proliferation, migration, invasion, and epithelial-mesenchymal transition (EMT) process in HSCC cell line FaDu. Most importantly, we illustrated that LEF1-AS1 played as a competitive endogenous RNA (ceRNA) via sponging miR-221-5p and thereby positively regulated gap junction protein alpha 1 (GJA1) expression, thus aggravated tumor progression and EMT. In conclusion, for the first time, we demonstrated lncRNA LEF1-AS1 as a novel biomarker for HNSCC and suggested LEF1-AS1/miR-221-5p/GJA1 axis as promising diagnostic and therapeutic target for HSCC treatment.

## 1. Introduction

Although only accounts for ~3–5% of all kinds of HNSCC, hypopharyngeal squamous cell carcinoma (HSCC) is one of the most lethal malignancies due to the grievous mortality (5 years overall survival rate less than 30% in late-stage patients) [1]. Because of the insidious onset, rapid development, and metastasis susceptibility, most patients (70% ~80%) with HSCC were at advanced stage when diagnosed, thus missed the optimal timing for surgical treatment [2]. Therefore, it is necessary to thoroughly elucidate the precise mechanisms of HSCC.

Recently, long noncoding RNAs (lncRNAs) which are identified as nonprotein-coding RNAs with over 200 nucleotides length have been revealed to play pivotal roles in molecular diagnosis and pathogenesis in almost all kinds of diseases including tumorigenesis and metastasis of cancers [3–5]. In HSCC, it has been proven that lncRNA HOXA11-AS contributed to the proliferation and migration via sponging and negatively regulation of miR-155 [6]. Moreover, lncRNA MALAT1 was found overexpressed in HSCC tissues and sponged miR-429 to stabilize the ZEB1 expression, resulting in the promotion of HSCC progression [7]. lncRNA AB209630 was shown to be decreased in HSCC tissues and identified as a suppressor of HSCC via inhibition of proliferation, invasion, metastasis, and survival of FaDu cells [8]. However, the functions of other lncRNAs in HSCC and potential mechanisms still remain unclear.

In the current manuscript, we illustrated the effect of lncRNA lymphoid enhancer-binding factor 1 antisense RNA 1 (LEF1-AS1) on HSCC. Bioinformatic analysis indicated that the increased expression of LEF1-AS1 in HNSCC tumors which was associated with the poor prognosis. Furthermore, we illustrated that LEF1-AS1 promoted HSCC cell proliferation, migration, invasion, and EMT process, whereas repressed cell apoptosis in both gain/loss-of-function experiments. Mechanically, LEF1-AS1 was shown to serve as a sponge for miR-221-5p to stabilize GJA1 levels. Our findings identified the oncogenic LEF1-AS1/miR-221-5p/GJA1 axis for HSCC progression and metastasis and suggested the axis as a promising target for HSCC therapy.

## 2. Materials and Methods

### 2.1. Bioinformatic Analysis

The transcriptome expression profile of HNSCC in The Cancer Genome Atlas (TCGA) database (https://cancergenome.nih.gov/) was collected. Analysis and graphics were created by *R* software (3.6.3). RNA22 (https://cm.jefferson.edu/rna22/Precomputed/) was applied to predict potential miRNA targets of LEF1-AS1, and TargetScan (http://www.targetscan.org/) was applied to seek for the candidate targets of miR-221-5p. Differentially expressed genes (DEGs) in LEF1-AS1 high or low expressed groups based on TCGA data were identified, and GSEA was performed using the Hallmark gene set (v.7.2) from MSigDB (https://www.gsea-msigdb.org/gsea/). Adjust *p* value<0.05, FDR < 0.25, and ∣NES | >1 were considered significant enrichment.

### 2.2. Cell Culture and Transfection

Human HSCC cell line FaDu was purchased from Chinese Academy of Sciences (Shanghai, China), and the cells were cultured in DMEM medium containing 10% FBS. The siRNA-NC, siRNA-LEF1-AS1, NC mimics, NC inhibitors, miR-221-5p mimics, and miR-221-5p inhibitors were all purchased from Genepharma (Shanghai, China) for transfection by using Lipofectamine RNAiMAX reagent (Thermo Fisher Scientific).

### 2.3. Proliferation Analysis and Apoptosis Analysis

The proliferation of FaDu cells was detected by CCK8 kits (Dojindo Laboratory, Japan). Briefly, the cells were seeded into 96-well plates and cultured for the indicated time, followed by the administration of CCK8 and incubation for another 4 h, and the absorption at 450 nm was examined by microplate reader (BioTek, San Diego, CA, USA). For colony formation, the transfected cells were counted, and 1000 cells were seeded into 6-well plates. 15 days later, the cell colonies were fixed with 4% paraformaldehyde and stained followed by the calculation. For cell apoptosis examination, after transfection for 48 h, the FaDu cell apoptosis was examined by using Annexin V-FITC/PI Kit (Beyotime, Shanghai, China) according to the manufacturer's instructions and detected by a flow cytometer.

### 2.4. Metastasis Analysis

For wound scratch assay, after transfection, scratch wounds were produced on the surface of overgrown cells by micropipette tip. After 24 h, the scratches were photographed, and the relative migrative rate was calculated by ImageJ. Basic transwell chamber (Corning) or Matrigel (BD Biosciences) precoated transwell chamber was used for the cell migration or invasion evaluation. Transwell assays were performed as previous described [9].

### 2.5. Quantitative Real-Time PCR (qRT-PCR) and RNA Immunoprecipitation (RIP) Assay

RNAs from FaDu cells were extracted by RNA Purification Kit (GeneJET; Thermo), and 1 *μ*g total RNA was reversely transcribed by cDNA synthesis Kit (TaKaRa, Dalian, China) followed by qRT-PCR carried out by SYBR Green PCR Mix (TaKaRa). Results were standardized to GAPDH or U6, and the fold changes were calculated by the 2^−*ΔΔ*CT^ method [10]. The sequences of primers were shown in Table 1. Magna RIP Kit (Millipore, USA) was used for RIP assay, the cell lysates were incubated with Ago2 antibody or negative control IgG antibody precoated beads (Millipore), and the purified RNA was performed the following qRT-PCR analysis.

### 2.6. Dual-Luciferase Reporter Gene Assay

The WT or the mutant-type of LEF1-AS1 or GJA1 was inserted into pmirGLO dual-luciferase vector, respectively (Promega, Madison, WI). FaDu cells were transfected with these vectors, together with the treatment of negative control or miR-221-5p mimics. 48 h later, the activities of luciferase were examined using the dual-luciferase reporter analysis system (Promega, Madison, WI, USA).

### 2.7. Western Blot Analysis

After transfection, FaDu cells were harvested with RIPA Lysis reagent (Sigma-Aldrich), and protein concentrations were assessed by BCA protein assay kit (Thermo Fisher). 20 *μ*g proteins were performed 12% SDS-PAGE then electrophoretically transferred onto PVDF membrane followed by blocking in 1% BSA. The membranes were incubated with individual antibodies overnight at 4°C. After incubating with the corresponding horseradish peroxidase- (HRP-) conjugated secondary antibodies, the band signals were measured by using enhanced chemiluminescence kit (Thermo Fisher Scientific). Antibodies for cleaved caspase 3 (#ab2302; 1 : 500), total caspase 3 (#ab32150; 1 : 1000), GAPDH (#ab8245; 1 : 1000), N-cadherin (#ab245117; 1 : 1000), and GJA1 (#ab217676; 1 : 1000) were obtained from Abcam, and antibodies for Bax (#89477; 1 : 1000), Bcl-2 (#15071; 1 : 1000), E-cadherin (#14472; 1 : 1000), and vimentin (#5741; 1 : 1000) were purchased from Cell Signaling Technology. Appropriate HRP-tagged secondary antibodies (1 : 2000) were all bought from Santa Cruz Biotechnology.

### 2.8. Statistical Analysis

Statistical analysis was performed by using GraphPad Prism 8.0 (GraphPad, USA) with student's *t*-test and one-way ANOVA together with Tukey Kramer post-hoc testing. *p* values <0.05 were considered statistically significant.

## 3. Results

### 3.1. lncRNA LEF1-AS1 Acts as a Novel Biomarker for HNSCC

**Figure 1:**
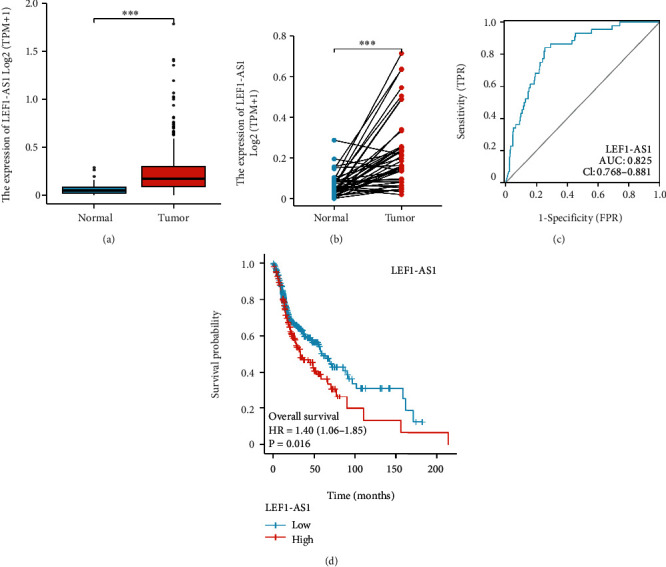
LncRNA LEF1-AS1 acts as a novel biomarker for HSCC. (a) The expression level of LEF1-AS1 in HNSCC tumor tissues (*n* = 502) and normal tissues (*n* = 44) from the TCGA database. (b) Comparison of the expression of LEF1-AS1 between tumor (*n* = 43) and matched normal tissues (*n* = 43) from the TCGA database. (c) ROC curve showed the diagnostic value of LEF1-AS1. (d) Kaplan–Meier curves revealed overall survival of HNSCC patients with high or low levels of LEF1-AS1 in TCGA. ^∗∗∗^*p* < 0.001.

To illustrate the effect of LEF1-AS1 on HNSCC, we performed the bioinformatic analysis in TCGA datasets and explored the expression and prognosis effect of LEF1-AS1 at first. As shown in Figures 1(a) and 1(b), we observed that the LEF1-AS1 expression was obviously elevated in HNSCC tumor tissues. Furthermore, ROC data suggested LEF1-AS1 as a potential biomarker for HNSCC (Figure 1(c)). Kaplan−Meier survival analysis also indicated that aggravated LEF1-AS1 levels were associated with poorer overall survival (OS) rate in HNSCC patients (Figure 1(d)). Due to the extremely low number of HSCC cases in the TCGA database, and HSCC and other HNSCC were all squamous cell carcinomas which shared similar pathological forms, and we speculated that LEF1-AS1 may also play crucial roles in HSCC tumorigenesis and development.

### 3.2. Effect of LEF1-AS1 on Cell Proliferation and Apoptosis

**Figure 2:**
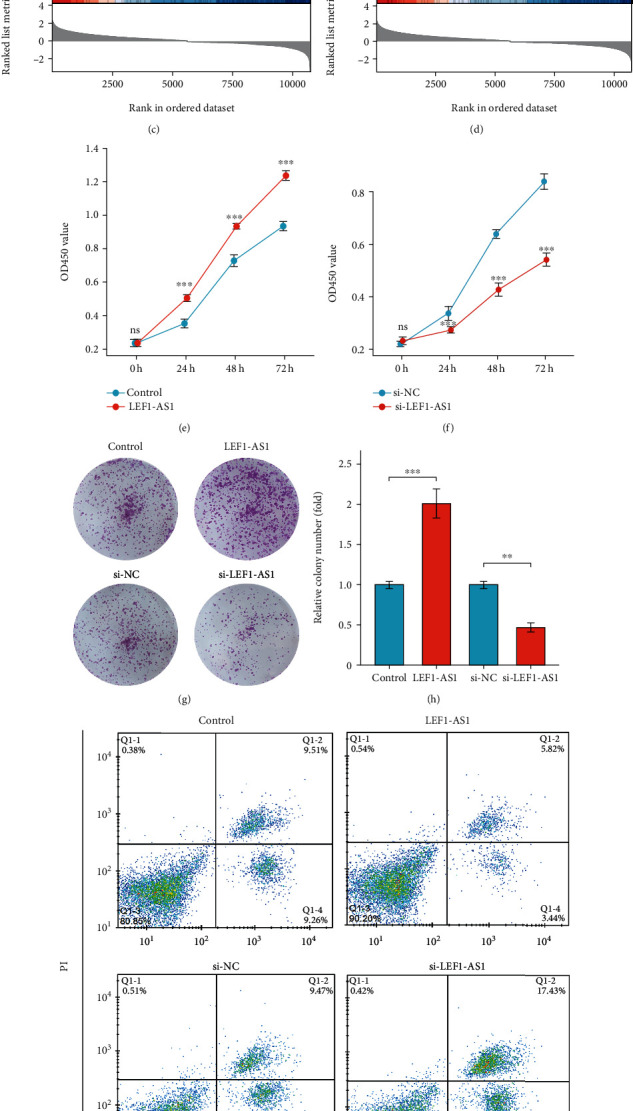
Effect of LEF1-AS1 on cell proliferation and apoptosis. (a, b) Efficiency of LEF1-AS1 overexpression plasmid (a) or LEF1-AS1 siRNAs (b) in FaDu cells. (c, d) GSEA analysis of P53 (c) and apoptosis (d) gene sets based on LEF1-AS1 expression information in TCGA. (e, f) CCK8 analysis in LEF1-AS1 overexpressed (e) or silenced FaDu cells (f). (g) Colony formation assay. (h) Relative colony numbers in (g). (i) Apoptosis of LEF1-AS1 overexpressed or silenced FaDu cells were detected by flow cytometry. (j) Apoptotic cell percent in (i). (k) Protein levels of apoptosis markers including cleaved caspase 3, Bax, and Bcl-2 in LEF1-AS1 overexpressed or silenced FaDu cells. Data are presented as means ± SD. ^∗∗^*p* < 0.01; ^∗∗∗^*p* < 0.001.

According to the abnormal expression and prognostic value of LEF1-AS1 in HNSCC datasets of the TCGA database, we further used HSCC cell line FaDu cells to examine the effect of LEF1-AS1 on HSCC tumorigenesis abilities. We used LEF1-AS1 overexpression plasmid and siRNAs for the gain/loss-of-function experiments, and the efficiency of the overexpression or silencing of LEF1-AS1 was detected by qPCR (Figures 2(a) and 2(b)). Gene set enrichment analysis (GSEA) results showed that LEF1-AS1 associated genes were enriched in p53 pathway (Figure 2(c)) and apoptosis process (Figure 2(d)). By using CCK8 experiment, we noticed that the LEF1-AS1 overexpression obviously increased the viability of FaDu cells (Figure 2(e)), and consistent results were obtained in LEF1-AS1 silenced cells (Figure 2(f)). The overexpression of LEF1-AS1 also induced elevated colony numbers, whereas silencing of LEF1-AS1 alleviated the formation of colonies (Figures 2(g) and 2(h)). Flow cytometry analysis revealed that LEF1-AS1 contributed to the suppression of cell apoptosis (Figures 2(i) and 2(j)), as well as the altered expression of apoptosis markers such as cleaved caspase 3, Bax, and Bcl-2 (Figure 2(k)).

### 3.3. LEF1-AS1 Positively Regulates Cell Migration, Invasion, and EMT Process

**Figure 3:**
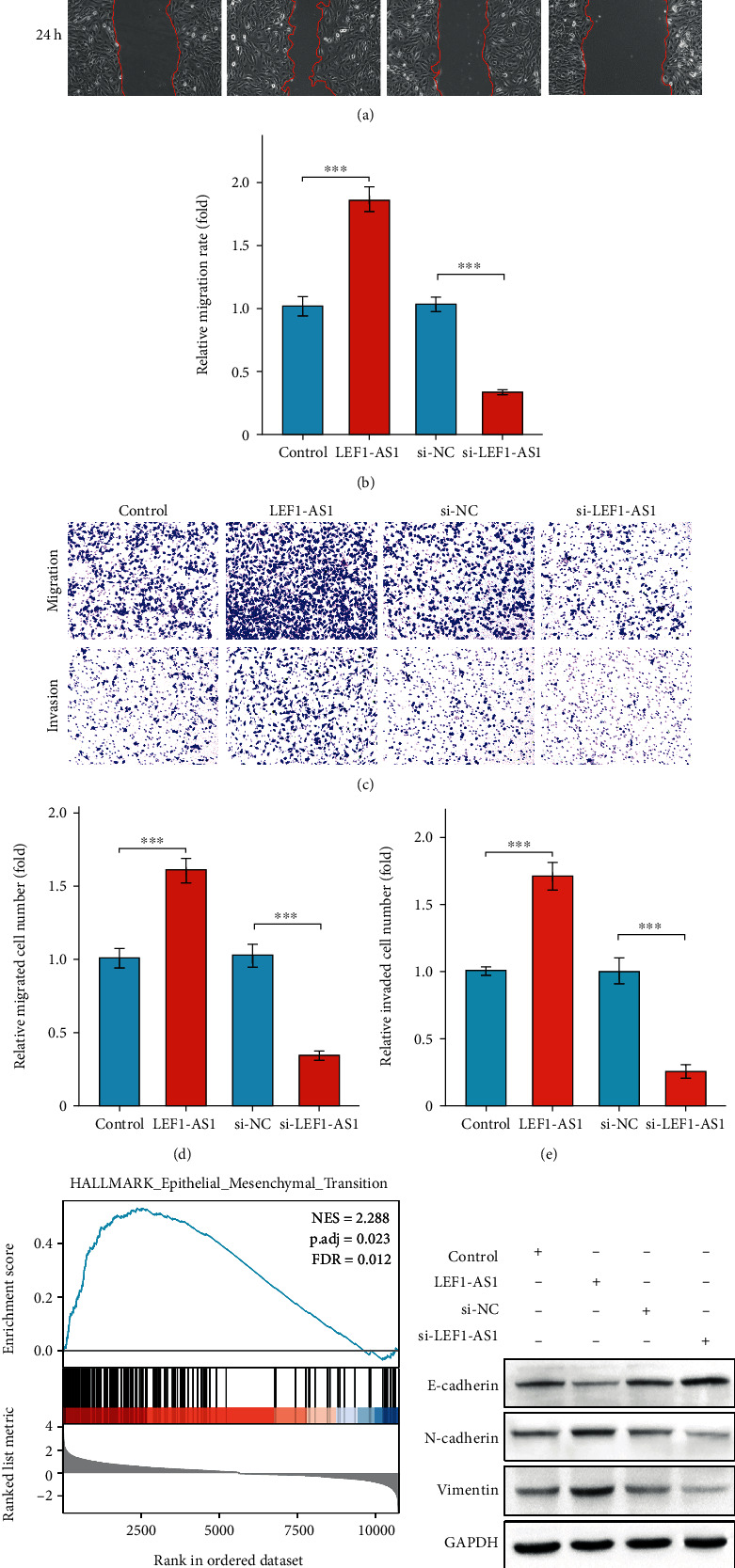
LEF1-AS1 positively regulates cell migration, invasion, and EMT process. (a) Wound healing assays in LEF1-AS1 overexpressed or silenced FaDu cells. (b) Relative migration rate in (a). (c) Transwell experiments for cell migration and invasion detection. (d, e) Relative migrated (d) or invaded (e) FaDu cell number in (c). (f) GSEA analysis of EMT gene sets based on LEF1-AS1 expression information in TCGA. (g) Western blot analysis of E-cadherin, N-cadherin, and vimentin in LEF1-AS1 overexpressed or silenced FaDu cells. Data are presented as means ± SD. ^∗∗∗^*p* < 0.001.

Furthermore, we examined the effect of LEF1-AS1 on tumor metastasis. As shown in Figures 3(a) and 3(b), wound scratch assay results indicated that cell migration ability was enhanced by the LEF1-AS1 overexpression, whereas silencing of LEF1-AS1 significantly suppressed FaDu migration. Moreover, we found that LEF1-AS1 contributed to cell migration and invasion in transwell experiments (Figures 3(c)–3(e)). Due to the critical role of EMT process in metastasis, we also performed GSEA enrichment of Hallmark EMT-related gene set, and we found that LEF1-AS1 was obviously correlated with EMT-related genes (Figure 3(f)), and western blot data indicated that LEF1-AS1 suppressed the E-cadherin expression, whereas elevated the expression of N-cadherin and vimentin (Figure 3(g)). In brief, these data indicated that LEF1-AS1 promotes HSCC cell migration and invasion and enhanced the process of EMT.

### 3.4. LEF1-AS1 Serves as ceRNA to Regulate the miR-221-5p Expression

**Figure 4:**
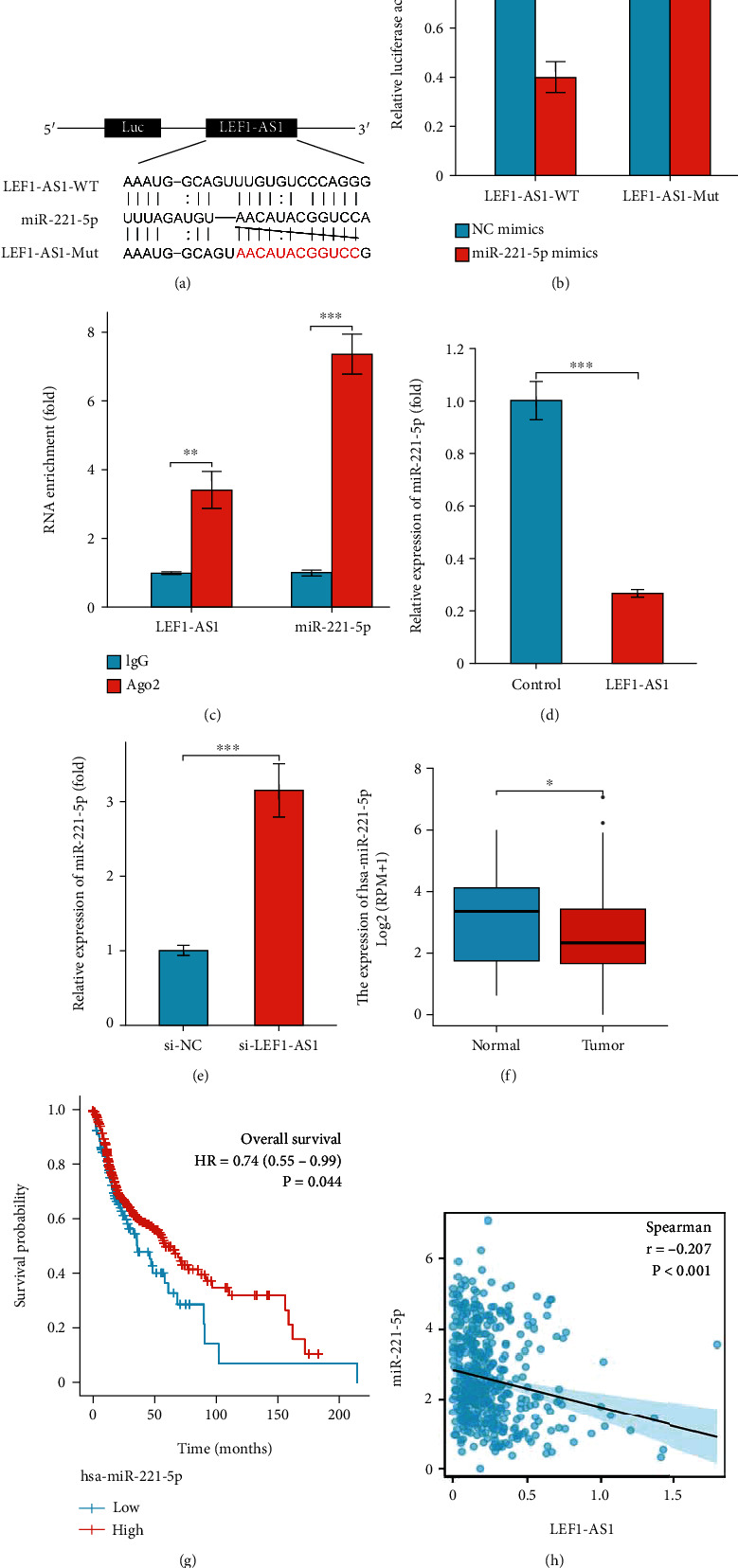
LEF1-AS1 acts as ceRNA to regulate the miR-221-5p expression. (a) Presentation of binding sites between LEF1-AS1 and miR-221-5p by RNA22 online tool. (b) Luciferase activity of LEF1-AS1-WT or LEF1-AS1-Mut plasmid in miR-221-5p overexpressed FaDu cells. (c) Anti-Ago2 RIP assay confirmed the combination between LEF1-AS1 and miR-221-5p. (d, e) Relative expression of miR-221-5p in LEF1-AS1 overexpressed (d) or silenced (e) FaDu cells. (f) The expression level of miR-221-5p in HNSCC tumor tissues (*n* = 525) and normal tissues (*n* = 44) from the TCGA database. (g) Kaplan–Meier curves revealed overall survival of HNSCC patients with high or low levels of miR-221-5p in TCGA. (h) Negative correlation between LEF1-AS1 and miR-221-5p in TCGA data. Data are presented as means ± SD. ^∗^*p* < 0.05; ^∗∗^*p* < 0.01; ^∗∗∗^*p* < 0.001.

Competing endogenous RNA (ceRNA) is one of the most essential functional mechanisms of lncRNA [11]. To investigate whether LEF1-AS1 regulates tumor progression and metastasis through ceRNA mechanism, we predicted the potential candidate targets of LEF1-AS1 by using online-tool RNA22, and we identified that LEF1-AS1 existed complementary binding regions to miR-221-5p (Figure 4(a)). Dual-Luciferase reporter gene assay data demonstrated that the miR-221-5p overexpression greatly suppressed the luciferase activation of LEF1-AS1-WT plasmid but failed to repress LEF1-AS1-mutant vector luciferase activity (Figure 4(b)). To substantiate this binding relationship, we further performed RIP experiment with anti-Ago2 in FaDu cells, and the enrichment effects of LEF1-AS1 or miR-221-5p together with Ago2 were confirmed (Figure 4(c)). Moreover, the levels of miR-221-5p were significantly suppressed in LEF1-AS1 overexpressed FaDu cells, whereas silencing of LEF1-AS1 elevated miR-221-5p levels obviously (Figures 4(d) and 4(e)). In addition, miR-221-5p was observed to be downregulated in HNSCC tumor tissues in TCGA-HNSCC datasets (Figure 4(f)). Furthermore, miR-221-5p was found to be positively correlated with the favorable overall survival of patients (Figure 4(g)), and miR-221-5p levels were shown to be negatively correlated with the LEF1-AS1 expression (Figure 4(h)). In brief, these data illustrated that LEF1-AS1 negatively regulates the miR-221-5p expression in FaDu cells.

### 3.5. miR-221-5p Suppresses FaDu Cell Proliferation and EMT by Targeting GJA1

**Figure 5:**
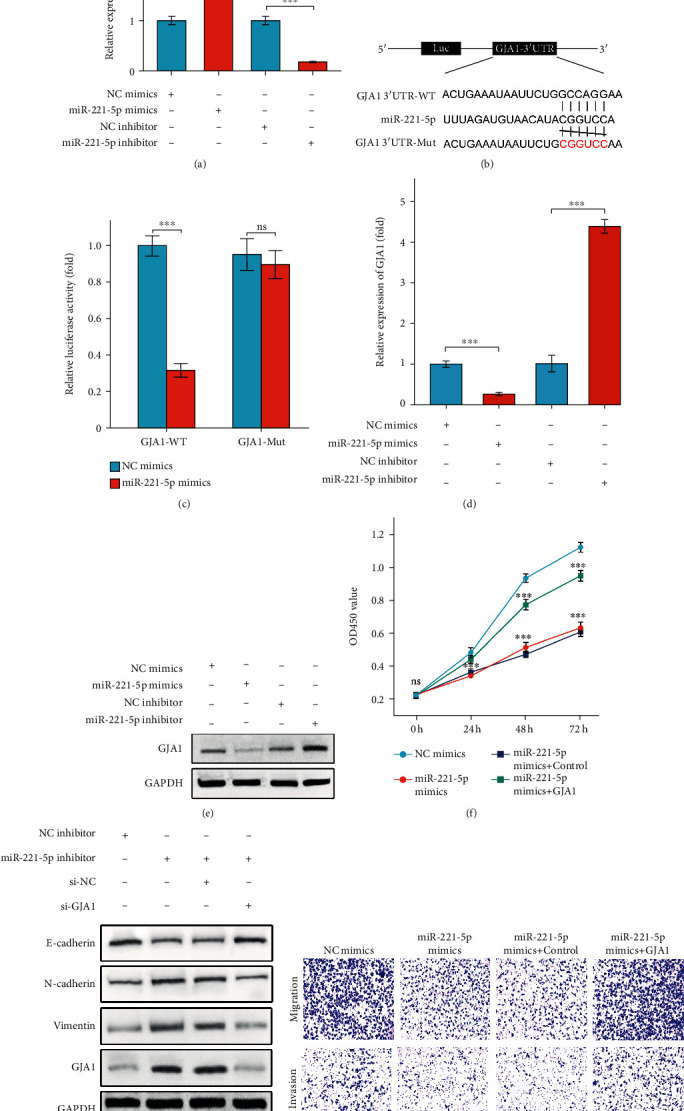
miR-221-5p suppresses FaDu cell proliferation and EMT by targeting GJA1. (a) Efficiency of miR-221-5p mimics and inhibitors. (b) Presentation of binding sites between miR-221-5p and GJA1 by TargetScan online tool. (c) Luciferase activity of GJA1-3′UTR-WT or GJA1-3′UTR-mutant plasmid in miR-221-5p overexpressed FaDu cells. (d, e) mRNA (d) or protein (e) levels of GJA1 in miR-221-5p overexpressed or silenced FaDu cells. (f) CCK8 results indicated that the GJA1 overexpression reversed miR-221-5p-induced inhibitory effect on cell proliferation. (g) Western blot results revealed that GJA1 silencing abolished miR-221-5p inhibitor-induced aggravation of EMT. (h) In transwell assays, the GJA1 overexpression reversed miR-221-5p-induced inhibitory effect on FaDu cell migration and invasion. Data are presented as means ± SD. ^∗∗∗^*p* < 0.001.

As shown in Figure 5(a), the efficiencies of miR-221-5p mimics and inhibitor were detected by qRT-PCR. For searching the putative targets of miR-221-5p, the TargetScan database was used which suggested GJA1 as a potential target (Figure 5(b)). Cotransfection of GJA1-3′UTR-WT vector together with miR-221-5p mimics resulted in an attenuated dual luciferase activity, whereas miR-221-5p mimics had no affection on luciferase activity of GJA1-3(h)UTR-mutant plasmid (Figure 5(c)). Furthermore, we observed that the miR-221-5p overexpression suppressed GJA1 levels, and miR-221-5p inhibition aggravated GJA1 expression significantly (Figures 5(d) and 5(e)). In addition, miR-221-5p was shown to inhibit FaDu cell proliferation, whereas rescued the levels of GJA1 abolished the inhibitory effect of miR-221-5p on proliferation (Figure 5(f)). Consistently, inhibitory functions of miR-221-5p on EMT, cell migration, and cell invasion were all reversed by GJA1 rescue experiments (Figures 5(g) and 5(h)). In brief, these data demonstrated that miR-221-5p attenuated FaDu cell proliferation and EMT process via targeting GJA1.

### 3.6. LEF1-AS1 Enhances the Growth and Metastasis Abilities of FaDu Cells via the miR-221-5p/GJA1 Axis

**Figure 6:**
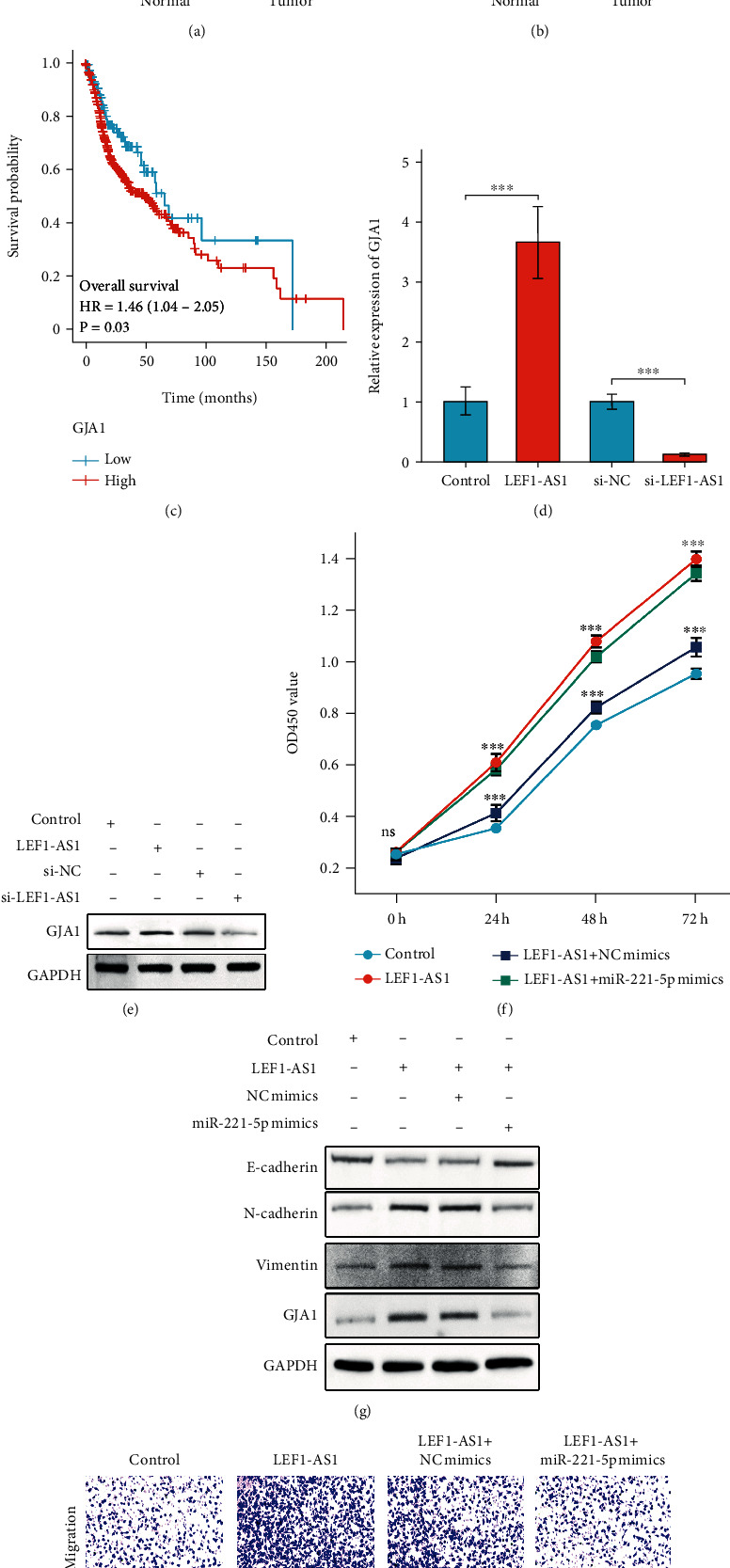
LEF1-AS1 enhances the proliferation and metastasis abilities of FaDu cells via the miR-221-5p/GJA1 axis. (a) The expression level of GJA1 in HNSCC tumor tissues (*n* = 502) and normal tissues (n =44) from the TCGA database. (b) Comparison of the expression of GJA1 between tumor (*n* = 43) and matched normal tissues (*n* = 43) from the TCGA database. (c) Kaplan–Meier curves revealed overall survival of HNSCC patients with high or low levels of GJA1 in TCGA. (d, e) mRNA (d) or protein (e) levels of GJA1 in LEF1-AS1 overexpressed or silenced FaDu cells. (f) CCK8 results indicated that the miR-221-5p overexpression reversed LEF1-AS1-induced promotion of cell proliferation. (g) Western blot results revealed that the miR-221-5p overexpression abolished LEF1-AS1-induced aggravation of EMT as well as the increased GJA1 expression. (h) In transwell assays, the miR-221-5p overexpression reversed LEF1-AS1-induced aggravation of FaDu cell migration and invasion. Data are presented as means ± SD. ^∗∗∗^*p* < 0.001.

Bioinformatic analysis revealed that the GJA1 expression was enhanced in HNSCC tumor tissues (Figures 6(a) and 6(b)), and Kaplan–Meier survival data demonstrated that the higher GJA1 expression presented a worse OS rate in HNSCC patients (Figure 6(c)). Moreover, we noticed that LEF1-AS1 dramatically enhanced the GJA1 expression (Figures 6(d) and 6(e)). Importantly, we observed that LEF1-AS1-induced FaDu cell proliferation and EMT were all reversed after transfection of miR-221-5p mimics, which mainly associated with the attenuated expression of GJA1 regulated by miR-221-5p (Figures 6(f)–6(h)). In brief, these data revealed that LEF1-AS1 acted as a ceRNA to stabilize the GJA1 expression via competing miR-221-5p, therefore, enhances the growth and metastasis abilities of FaDu cells.

## 4. Discussion

In the current manuscript, we demonstrated the functions of lncRNA LEF1-AS1 in HSCC and illustrated the prooncogenic effect of LEF1-AS1 via promoting the tumor progression and metastasis. As far as we known, this manuscript is the first publication about the functions of LEF1-AS1 in HSCC and verified the role of the LEF1-AS1/miR-221-5p/GJA1 axis.

In recent time, accumulating evidences suggested that the overwhelming majority of lncRNA function as molecular sponges for miRNAs to weaken the expression of miRNAs, therefore indirectly regulated miRNAs targets levels in diverse kinds of diseases. For instance, lncRNA BCRT1 was reported to competitively bind with miR-1303 to prevent the degradation of PTBP3, which induced the progression of breast cancer [12]. In LPS-induced HK2 cells, lncRNA NKILA was observed to aggravate LPS-induced apoptosis and inflammation via miR-140-5p sponging-associated stabilization of CLDN2 [13]. The lncRNA-PVT1/miR-619-5p/Pygo2/ATG14 axis was shown to be critical for the promotion of gemcitabine chemoresistance of pancreatic cancer [14]. The roles of LEF1-AS1 have been illustrated in other diseases in previous studies. As examples, the LEF1-AS1 expression could be induced by CREB1, and the high expression of LEF1-AS1 promoted tumorigenesis of colorectal tumor through sponging miR-489 and stabilizing DIAPH1 [15]. Moreover, LEF1-AS1 was found to aggravate the progression of ovarian cancer [16], retinoblastoma [17], and lung cancer [18]; however, the effect of LEF1-AS1 on HNSCC, especially in hypopharyngeal squamous cell carcinoma, still remains largely unknown. In this research, we illustrated the function of LEF1-AS1 in HSCC. We observed that LEF1-AS1 was upregulated in tumor tissues in the TCGA database, which was correlated with the poor prognosis. In vitro experiments revealed that LEF1-AS1 significantly enhanced cell proliferation as well as the suppression of cell apoptosis. Moreover, LEF1-AS1 was proved to enhance EMT process and improved metastasis of HSCC cells. Mechanically, it was found that LEF1-AS1 served as a sponge of miR-221-5p thereby alleviated miR-221-5p induced decreased levels of GJA1.

Antitumor effects of miR-221-5p have been illustrated previously. Jiang and colleagues demonstrated that the miR-221-5p expression was suppressed in gastric cancer tissues, overexpression of miR-221-5p reduced cisplatin chemoresistance of gastric tumor cells, and suppressed cell proliferation and migration via suppressing DDR1 [19]. Moreover, miR-221-5p was reported to inhibit prostate tumor cell proliferation and metastasis both in vivo and in vitro [20]. Consistently, we found that the miR-221-5p expression was reduced in tumor tissues in HNSCC datasets of TCGA which was negatively associated with LEF1-AS1 expression, and miR-221-5p was demonstrated as a tumor suppressor to inhibit cell growth and EMT-associated migration and invasion. By bioinformatical analysis, we identified GJA1 as the potential target of miR-221-5p. GJA1 was shown to be positively correlated with the poor overall survival of cervical cancer [21]. Moreover, GJA1 was shown to promote hepatocellular carcinoma progression via TGF-*β* activation and enhancement of EMT process [22]. Effect of GJA1 on proliferation and EMT ability was also confirmed in breast cancer [23], lung cancer [24], and bladder cancer [25]. Our results indicated that the GJA1 expression was increased in HNSCC tissues and correlated with the worse prognosis. In addition, we noticed that the GJA1 overexpression reversed miR-221-5p mimic-induced EMT inhibition and growth suppression, which confirmed the assumption that GJA1 was the target of miR-221-5p. At last, by performing rescue assays, we found that miR-221-5p mimic administration abolished LEF1-AS1-induced FaDu proliferation and EMT process and revealed that the function of LEF1-AS1 on FaDu progression and metastasis mainly depends upon the LEF1-AS1/miR-221-5p/GJA1 axis.

## 5. Conclusion

In summary, our manuscript suggested LEF1-AS1 as a novel biomarker for HNSCC, illustrated the effects of LEF1-AS1, miR-221-5p, and GJA1 on hypopharyngeal squamous cell carcinoma for the first time, and revealed that the LEF1-AS1/miR-221-5p/GJA1 axis may serve as a novel promising target for HSCC therapy. However, further exploration about the precise mechanisms by which GJA1 regulates EMT process and cell proliferation in HSCC is needed in the further studies.

## Figures and Tables

**Table 1:** Sequences of primers used in the study.

Gene	Sequence (5′-3′)
LEF1-AS1	
F	AAG GAC GAG AGA AAA GCA C
R	CAC ACA AAG GGG AAG ACC
GAPDH	
F	GTC TCC TCT GAC TTC AAC AGC G
R	ACC ACC CTG TTG CTG TAG CCA A
miR-221-5p	
F	ACACTCCAGCTGGGACCTGGCATACAATGT
R	CTC AAC TGG TGT CGT GGA
GJA1	
F	GGA GAT GAG CAG TCT GCC TTT C
R	TGA GCC AGG TAC AAG AGT GTG G
U6	
F	CTC GCT TCG GCA GCA CA
R	AAC GCT TCA CGA ATT TGC GT

## Data Availability

The data used and analyzed during the current study are available from the corresponding author on reasonable request.
